# Magnetic-controlled capsule endoscopy performance in aging patients

**DOI:** 10.1186/s12876-023-02914-0

**Published:** 2023-08-11

**Authors:** Jiaxin Li, Li Li, Yueyuan Li, Long Chen, Rongyue Liang, Meilin Liu, Hongmei Jiao

**Affiliations:** Department of Geriatrics, Peking University First Hospital, No. 8 Xishiku St., Xicheng District, Beijing, 100034 China

**Keywords:** Magnetic capsule endoscopy, Transit time, Visualization score, Cleanliness score

## Abstract

**Background:**

The increasing elderly population and wide use of magnetic capsule endoscopy (MCE) have led to more attention to elderly patients.

**Aim:**

The aim of this study was to assess the performance (including transit time, cleanliness score, positive findings and safety) of MCE in aging patients (≥ 60 years), especially patients over 80 years old.

**Methods:**

Consecutive patients of ≥ 60 years undergoing MCE at our center from August 2017 to August 2022 were classified into the oldest (≥ 80 years) and the older (60–79 years) groups. Esophageal transit time (ETT), gastric examination time (GET), small bowel transit time (SITT), and the quality of gastric preparation were compared. Information on examination indications, subjective discomforts, adverse events, and MCE outcomes were compared.

**Results:**

Of 293 enrolled patients, 128 patients were in the oldest group and 165 patients were in the older group. ETT and GET were longer in the oldest group, whereas SITT was slightly longer in the oldest patients. Visualization scores were significantly lower in the body and antrum in the oldest patients. The total visualization score was lower in the older group compared with the oldest group; however, the difference was not significant. Cleanliness scores at the fundus and antrum and total cleanliness scores were lower in the oldest patients compared with the older patients. Positive findings and ulcers and erosions in the small intestine were more common in the oldest group. One patient had nausea during the gastric examination. Capsule retention in the cecum occurred in one case.

**Conclusion:**

MCE was feasible and safe for aging patients. ETT and GET were markedly longer and gastric cleanliness and visualization were worse, while overall small intestine-positive findings were higher in the oldest patients compared with the older patients.

**Supplementary Information:**

The online version contains supplementary material available at 10.1186/s12876-023-02914-0.

## Introduction

Life expectancy has increased throughout most of the world, and the incidence of gastrointestinal diseases increases with increasing age. For example, peptic ulcers and gastric cancer occur more frequently in elderly patients [1]. Esophagogastroduodenoscopy (EGD) is an essential method for screening gastric diseases. However, the standard EGD requires the insertion of a flexible endoscopy, resulting in discomfort and poor compliance in elderly patients. Although conscious sedation improves the subjective endoscopy experiences of patients, many elderly patients have contraindications for sedation, and most published studies excluded patients over 80 years old.

Magnetic-controlled capsule endoscopy (MCE) was developed to completely visualize the stomach [2]. Due to its noninvasiveness and excellent diagnostic performance compared to conventional gastroscope, MCE has become increasingly popular [3, 4]. Recently, H. pylori infection status could be accurately assessed on MCE according to the Kyoto classification of gastritis [5]. However, gastric mucus, bubbles, and other residual debris obscure mucosal visualization [6–8]. Diagnosis of early gastric cancer can be improved with effective pre-medication that decreases mucus and bubbles during conventional EGD [9]. Therefore, adequate preparation of the mucosal surface is critical for non-invasive tests like MCE.

MCE can also be used to examine the small intestinal mucosa in a single examination. MCE is a well-established method for examining patients for both gastric and small intestinal diseases, such as gastric cancer, iron deficiency anemia, and melaena [10, 11]. The MCE system was approved by the National Medical Products Administration of China as an established investigation modality for gastric and small intestinal examinations in adults.

Gastric cleansing quality and visualization and the safety of cleansing methods were previously investigated mainly in healthy volunteers [6, 8, 12] and patients with upper gastrointestinal complaints [3, 12]. However, these methods have not been widely evaluated in special populations, especially patients over 80 years. The health, disease, and frailty of aging patients are heterogeneous compared with other adults.

We conducted a single-center retrospective study analyzing data from aging patients (≥ 60 years) who underwent MCE examinations of the stomach and small intestine. The quality of the gastric preparation, digestive tract transit time, efficiency, and MCE safety were evaluated. The results of this study can facilitate gastrointestinal tract examinations in aging patients, especially patients over 80 years old.

## Materials and methods

### Study design, setting, and patients

**Fig. 1:**
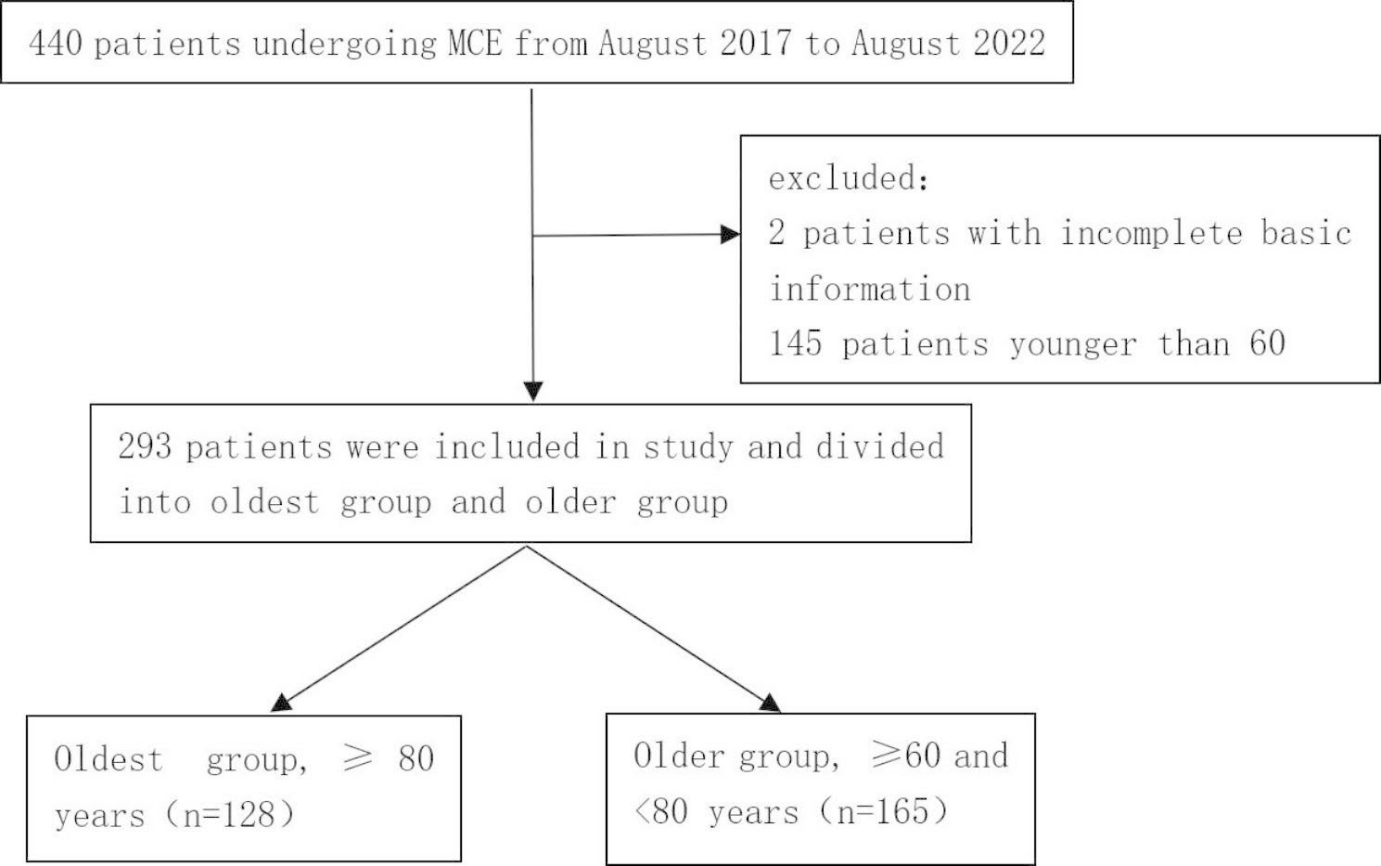
Schematic flow diagram of the study

This was a single-center, retrospective study. Patients with any of the following conditions were not allowed MCE examination: (1) dysphagia or symptoms of gastric outlet obstruction; (2) congestive heart failure, renal insufficiency or claustrophobia; (3) implanted metallic devices such as pacemakers, defibrillators, artificial heart valves or joint prostheses; (4) pregnancy. Data from patients at PKUFH (Peking University First Hospital) who underwent MCE examination from August 2017 to August 2022 were retrospectively analyzed. Exclusion criteria included (a) patients under 60 years of age, (b) incomplete basic information or imaging data. Patients were divided into an oldest group (≥ 80 years) and an older group (≥ 60 and < 80 years) (Fig. 1). The study was approved by the Medical Ethics Committee of Peking University First Hospital, and patient consent was waivered because all personally identifiable information was removed from the data sets.

### MCE examination procedure

**Fig. 2:**
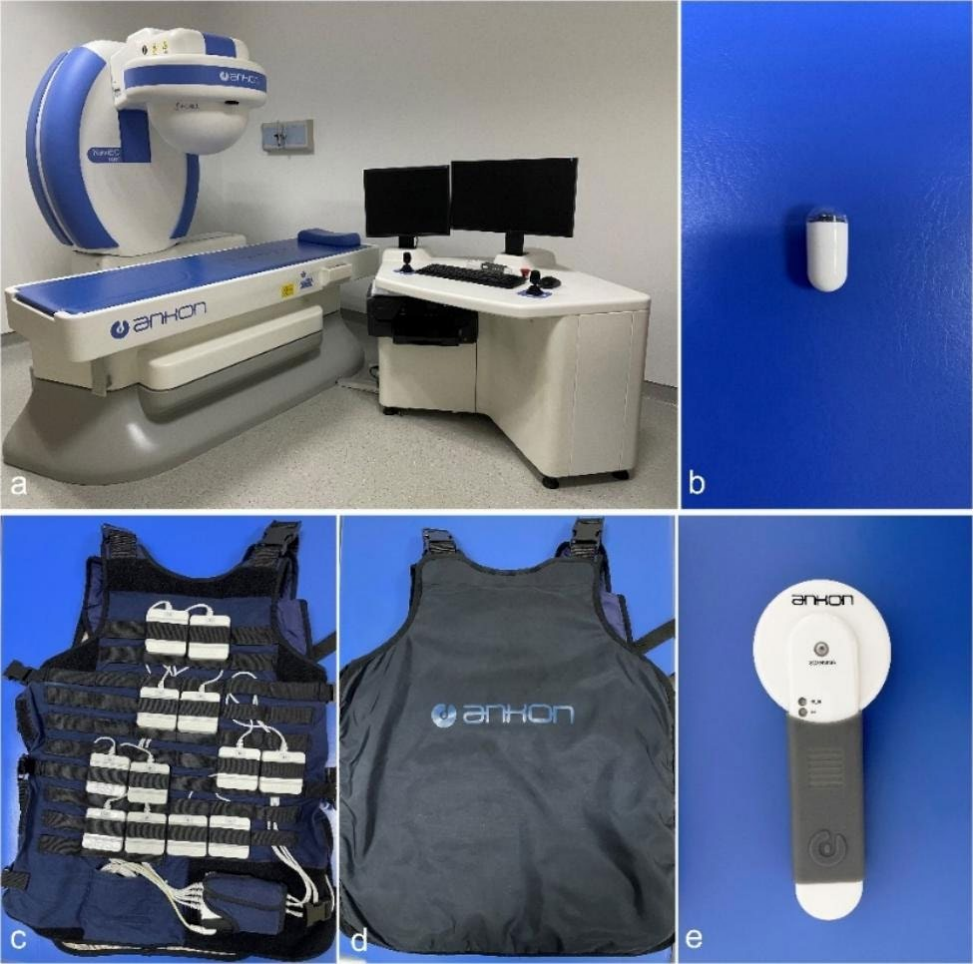
The MCE system, **(a)** Guidance magnetic robot and a computer workstation with ESNavi software, **(b)** Endoscopic capsule (11.8 × 27 mm), **c&d**. Data recorder (check suit), **e**. Detector

The MCE system (Ankon Technologies Co. Ltd., Wuhan, China) consisted of an endoscopic capsule (11.8 × 27 mm), a guidance magnetic robot, a data recorder (check suit), and a computer workstation with ESNavi software(Fig. 2). Patients took a clear liquid diet (supper) and fasted overnight (> 8 h). Gastric preparations with simethicone and pronase were administered to improve gastric cleanliness [8, 9]. Some patients undergoing small intestinal examinations also received 2 L of polyethylene glycol (PEG, Wanhe Pharmaceutical Co. Ltd., China) 12 h before the MCE and 1 L of PEG 3 h before the MCE.

Patients who underwent the standard gastrointestinal preparation regimen for MCE were instructed to swallow the capsule in the left lateral position with a small amount of water to facilitate esophageal passage. The operator inspected images of the entire esophagus in real time. After entering the stomach, the capsule was rotated and advanced to the fundus and cardiac regions, followed by the gastric body, angulus, antrum, and pylorus under magnetic control. This procedure was repeated twice to better visualize the gastric mucosa. The stomach was usually filled by ingesting 800–1000 mL of water during the gastric examination [10]. After completing the gastric examination, the capsule was dragged to the duodenum if the pylorus was open, and the duodenum was examined under magnetic control. If the pylorus was not open or the capsule could not pass through the pylorus under magnetic control, the capsule was switched to “small intestine mode” with an adaptive capture rate of 0.5–2 fps. Patients were allowed to leave the hospital with the data recorder for further image collection [13]. Patients were allowed to drink clear liquids and eat small amounts of solid food 2 h later.

Patient baseline information, including age, sex, body mass index (BMI), indications for MCE, and medical history, were retrospectively collected. Examinations were performed by two operators (L.L and J.L at PKUFH) who performed more than 200 MCEs. Patients were followed up for 2 weeks to confirm the capsule excretion. Each MCE video was independently and blindly interpreted by two other experienced gastroenterologists. In the case of a discrepancy in the interpretation of capsule findings between the two MCE readers, a central committee composed of two MCE experts was consulted to reach a final decision.

### Study outcomes and definition

#### Primary outcomes

The primary outcomes of this study included, esophageal transit time (ETT), gastric examination time (GET), small intestinal time (SITT) and visualization and cleanliness of the stomach. ETT defined as the times between the first esophageal image and the first gastric image. GET was defined as the time from the first gastric image to the end of the gastric examination under magnetic control. SITT meant the first small intestine image and the first large intestine image [13]. Pyloric transit time (PTT) was defined as the time from the completion of the gastric examination under magnetic control to the time the MCE entered the duodenum [13]. The ratio of the capsule reaching the cecum within 8 h was analyzed.

**Fig. 3:**
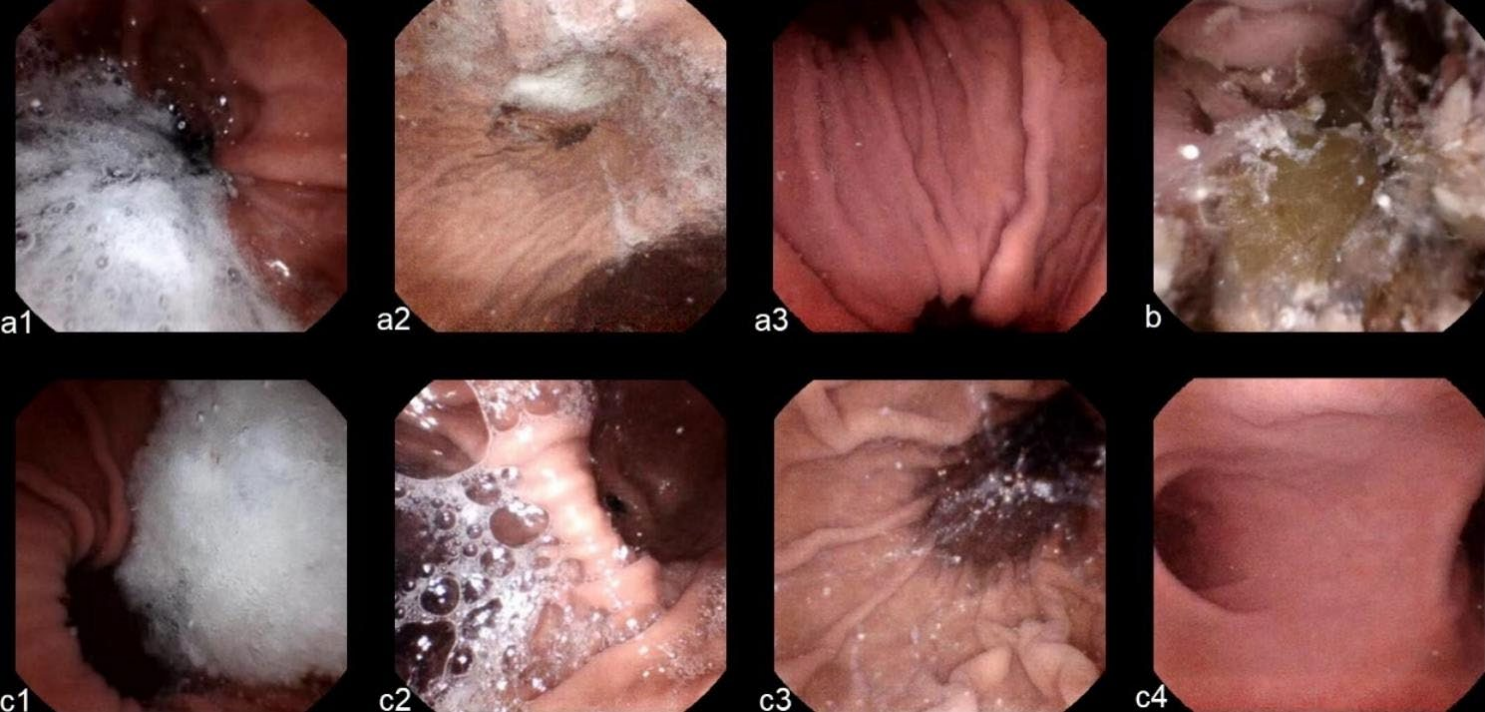
Representative images showing the 3-point grading scale for gastric visualization: **a1.** 1, poor, < 70% of the mucosa was observed; **a2.** 2, fair, 70–90% of the mucosa was observed; and **a3.** 3, good, > 90% of the mucosa was observed. **b.** Representative image of food debris. **c.** Representative images showing the 4-point grading scale for stomach cleanliness during magnetic capsule endoscopy. **c1.** 1, poor, large amount of mucus or foam residue; **c2.** 2, fair, considerable amount of mucus or foam present to preclude a completely reliable examination; **c3**. 3, good, small amount of mucus and foam, but not enough to interfere with the examination; **c4.** 4, excellent, no more than small bits of adherent mucus and foam

Gastric visualization and cleanliness, which are important for finding lesions, were compared. Gastric visualization (Fig. 3) included visualization of the gastric mucosa at 6 anatomic landmarks: cardia, fundus, body, angulus, antrum, and pylorus. A 3-point visualization score was used, as follows: 1, poor (< 70% of the mucosa was observed); 2, fair (70–90% of the mucosa was observed); and 3, good (> 90% of the mucosa was observed) [3]. The summation of the scores from the six regions was also calculated; higher scores indicated better visualization.

Gastrointestinal tract cleanliness is mainly affected by mucus and bubbles. Gastric cleanliness was scored using a 4-grade scale, as follows: 1, poor, large amount of mucus or foam residue; 2, fair, considerable amount of mucus or foam present precluding a completely reliable examination; 3, good, small amount of mucus and foam, but not enough to interfere with the examination; and 4, excellent, not more than small bits of adherent mucus and foam [9].

#### Secondary outcomes

Secondary outcomes included examination indications, positive findings in the stomach or small intestine, and safety. Positive findings included ulcers, erosions, angioectasia, bleeding points, hyperemia/erythema, polyps, and tumors [14]. Safety was assessed by the occurrence of procedure-related adverse events during a 2-week follow-up period, including abdominal pain, bleeding, nausea, and capsule retention. Capsule retention was checked by detector (Fig. 2e) scanning.

### Statistical analyses

Categorical data were compared using chi-squared tests or Fisher exact tests and presented as numbers (percentages). The Kolmogorov-Smirnov test was applied to assess the normal distribution of data. Normally distributed continuous data were compared using independent-sample t-tests and presented as means ± standard deviations (SDs). Non-normally distributed continuous data were compared using Mann-Whitney U-tests and presented as medians with interquartile ranges (IQRs). The intra-class correlation coefficient was used to analyze the consistency of gastric condition evaluation by the two endoscopists. A two-sided P-value < 0.05 was considered significant. Statistical analyses were performed using SPSS software (ver. 26.0, SPSS Inc., Chicago, IL, USA) and GraphPad Prism 8.0 (GraphPad Software, La Jolla, CA, USA).

## Results

### Patient characteristics

The 293 patients enrolled in the study included 128 patients in the oldest group and 165 patients in the older group. The 197 patients who completed gastric and small intestinal examinations included 98 patients in the oldest group and 99 patients in the older group. 81 (63.3%) of the oldest patients were 80–89 years, 46 (35.9%) were 90–96 years, and one was 100 years old. Sex, history of diabetes mellitus, and atrial fibrillation were not significantly different between the two groups. BMI was lower and the histories of coronary artery disease and antithrombotic therapy were more common in the oldest patients. The most common indication for MCE in the oldest patients was gastrointestinal injury assessment (27.3%), followed by dyspepsia (18.8%), GI bleeding or iron deficiency anemia (17.2%), physical examination (14.1%), poor appetite (6.3%), acid reflux (6.3%), elevated tumor markers for gastrointestinal malignancies (3.1%), and ulcers (2.3%). The proportions of dyspepsia, GI bleeding, iron deficiency anemia, and poor appetite were higher in the oldest group. Baseline characteristics and indications for MCE in the two groups are summarized in Table 1.

**Table 1:** Characteristics and indications for MCE in the two groups (n = 293)

	Oldest (n = 128, 43.7%)	Older (n = 165, 56.3%)	P-value
Age, median (IQR)	87.0 (84.0, 91.0)	65.0 (62.0,74.0)	P < 0.001
Male/female, n	109/19	127/38	0.079
BMI, mean ± SD, kg/m^2^	23.6 ± 3.4	24.8 ± 2.8	P = 0.005
Comorbidity			
Coronary artery disease, n (%)	49 (38.3)	43 (26.1)	P = 0.025
Atrial fibrillation, n (%)	11 (8.6)	10 (6.1)	P = 0.404
Diabetes mellitus, n (%)	36 (28.1)	35 (21.2)	P = 0.171
Antithrombotic therapy history, n (%)	81 (64.8)	78 (47.3)	P = 0.003
Indication for MCE			
GI bleeding/iron deficiency anemia, n (%)	22 (17.2)	13 (7.9)	P = 0.015
Dyspepsia, n (%)	24 (18.8)	58 (35.2)	P = 0.002
Ulcer, n (%)	3 (2.3)	5 (3.0)	P = 1.000
Poor appetite, n (%)	8 (6.3)	1 (0.6)	P = 0.012
Acid reflux, n (%)	8 (6.3)	8 (4.8)	P = 0.600
Elevated tumor markers for gastrointestinal malignancies, n (%)	4 (3.1)	2 (1.2)	P = 0.409
GI injury assessment, n (%)	35 (27.3)	42 (25.5)	P = 0.789
Physical examination. n (%)	18 (14.1)	25 (15.2)	P = 0.868
Others*, n (%)	4 (3.1)	5 (3.0)	P = 1.000

MCE, Magnetic-controlled capsule endoscopy; BMI, body mass index; GI, gastrointestinal. *Weight loss, cirrhosis, and lymph node enlargement

### Transit time and examination time in the elderly

The median ETT, and GET of MCE were longer in the oldest group compared with the older group (186.5 s [IQR, 46.8–312.3] vs. 75.0 s [IQR, 31.0–175.0], P = 0.001; 30.0 min [IQR, 25.0–35.0] vs. 27.0 min [IQR, 24.0–31.0], P = 0.003, respectively). Successful transpyloric passage of the MCE capsule using magnetic guidance was achieved in 76.6% (98/128) of patients in the oldest group and 80.6% (133/165) of patients in the older group (P = 0.401). No significant differences in PTT were detected between the two groups (3.0 min [IQR, 0.0–25.0] vs. 2.0 min [IQR, 0.0–24.0], P = 0.409).

In the small intestine, no significant differences in the SITT of MCE were detected between the two groups (357.0 min [IQR, 262.8–421.0] vs. 308.0 min [IQR, 233.0–395.0], P = 0.094). The completion rate to the cecum within 8 h was 61.2% (60/98) in the oldest group and 70.7% (70/99) in the older group (P = 0.160) (Table 2).

**Table 2:** Preparation and transit time in the two groups (n = 293)

	Oldest (n = 128, 43.7%)	Older (n = 165, 56.3%)	P-value
PEG, n (%)	21 (16.4)	31 (19.4)	P = 0.510
ETT, sec, median (IQR)	186.5 (46.8, 312.3)	75.0 (31.0, 175.0)	P = 0.001
GET, min, median (IQR)	30.0 (25.0, 35.0)	27.0 (24.0, 31.0)	P = 0.003
PTT, min, median (IQR)	3.0 (0.0, 25.0)	2.0 (0.0, 24.0)	P = 0.409
Transpyloric passage of MCE by magnetic guidance, n (%)	98 (76.6)	133 (80.6)	P = 0.401
SITT, min, median (IQR)	357.0 (262.8, 421.0)	308.0 (233.0, 395.0)	P = 0.094
Completion to the cecum within 8 h, n (%)	60 (61.2)	70 (70.7)	P = 0.160

PEG, polyethyleneglycol; ETT, esophageal transit time; GET, gastric examination time; PTT, pyloric transit time; SITT, small intestine transit time

### Gastric visualization and cleanliness score

**Fig. 4:**
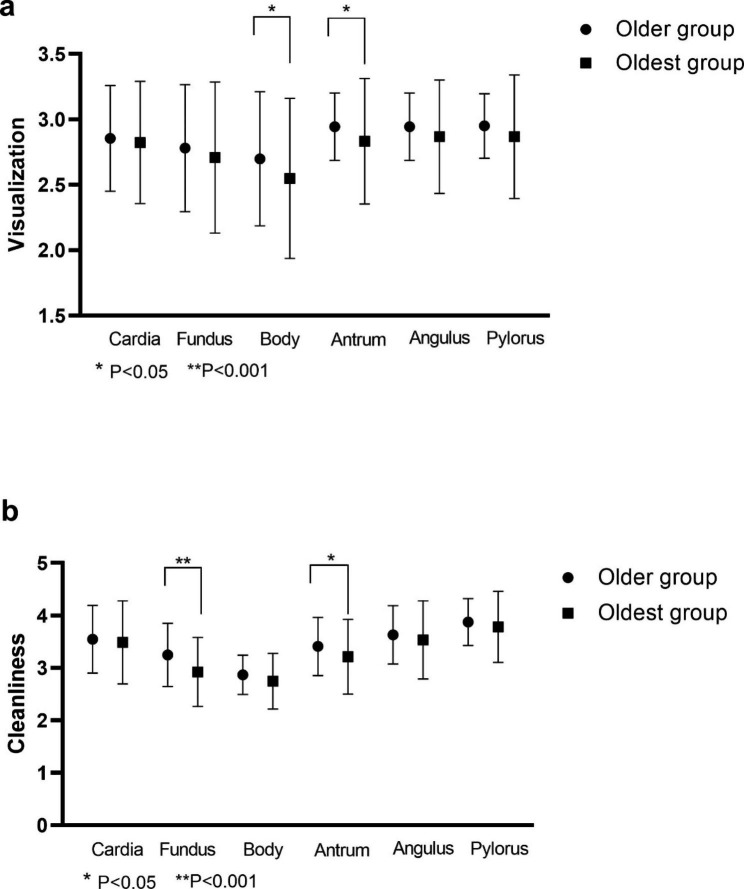
Gastric visualization and cleanliness scores in the two age groups. **(a)** Visualization scores were significantly lower in the body and antrum in the oldest patients compared to the older patients (P = 0.039 and P = 0.026). **(b)** The oldest patients showed lower cleanliness scores at the fundus and antrum compared with older patients (P < 0.001, P = 0.035, respectively)

The intra-class correlation coefficient, indicating the consistency between the two endoscopists in evaluating gastric total cleanliness score, was 0.867 (95% CI: 0.677–0.936, P < 0.001). Gastric visualization and cleanliness in the two groups are shown in Fig. 4. The visualization scores in the body and antrum of the stomach were significantly lower in the oldest group compared with the scores in the older group (P = 0.039 and P = 0.026). Total visualization scores were not significantly different between the two groups (18.0 (16.0,18.0) vs. 18.0 (17.0,18.0), P = 0.069). Cleanliness scores for the gastric fundus and antrum were significantly different between the two groups (P < 0.001 and P = 0.035, respectively). Total gastric cleanliness scores were 20.0 (IQR, 19.0–21.5) in the oldest group and 21.0 (IQR, 20.0–22.0) in the older group (P = 0.022).

### Positive findings and safety

**Fig. 5:**
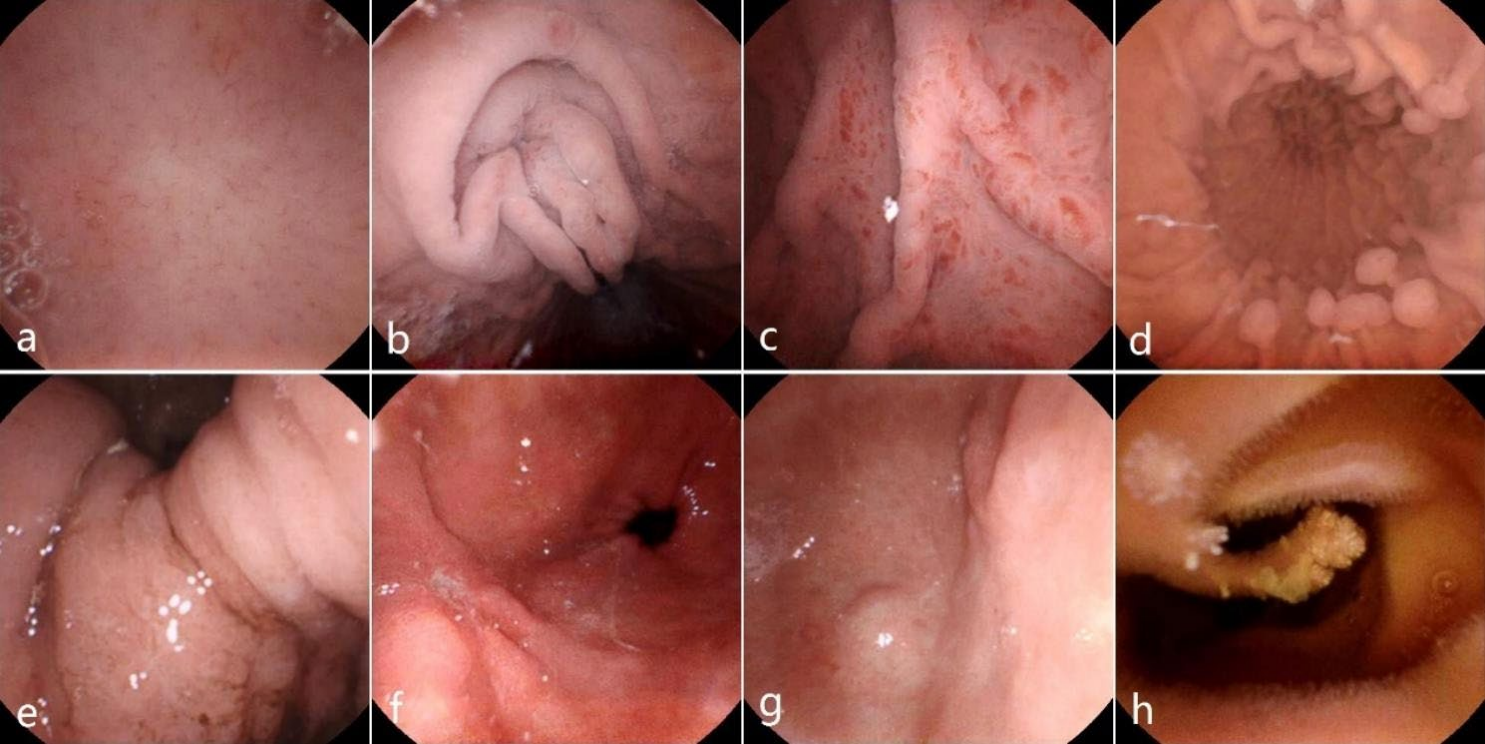
Positive findings, **(a)** Regular arrangement of collecting venules (RAC), **(b)** Cardiac inflammation, **(c)** Fundus spotty redness, **(d)** Body polyps, **(e)** Angulus tumor and bleeding, **(f)** Antrum ulcer, **(g)** Antrum tumor, **(h)** Small bowel polyp

The pathological lesions found during MCE are shown in Table 3 and Fig. 5. Positive findings in the stomach were higher in the oldest group compared with the older group but the differences were not significant (82.8% vs. 73.8%, P = 0.066). The incidences of small intestinal erosion and ulcers were higher in the oldest group compared with the incidences in the older group (20.8% vs. 10.7%, P = 0.032 and 21.7% vs. 7.6%, P = 0.002, respectively). No significant differences in the incidences of angioectasia, bleeding points, hyperemia/erythema, polyps, and tumor were detected between the two groups.

**Table 3:** Positive findings in the stomach and small intestine in the two groups (n = 293)

Lesions	Oldest (n = 128, 43.7%)	Older (n = 165, 56.3%)	P-value
Stomach, n (%)	106 (82.8)	121(73.8)	P = 0.066
Ulcer, n (%)	15 (11.7)	14 (8.5)	P = 0.358
Erosions, n (%)	27 (21.1)	36 (21.8)	P = 0.881
Angioectasia, n (%)	2 (1.6)	4 (2.4)	P = 0.699
Bleeding point, n (%)	18 (14.1)	17 (10.3)	P = 0.325
Hyperemia/erythema, n (%)	43 (33.6)	56 (33.9)	P = 0.951
Polyp, n (%)	45 (35.2)	44 (26.7)	P = 0.117
Tumor, n (%)	6 (4.7)	4 (2.4)	P = 0.341
The small intestine, n (%)	50 (47.2)	44 (33.6)	P = 0.034
Ulcer, n (%)	23 (21.7)	10 (7.6)	P = 0.002
Erosions, n (%)	22 (20.8)	14 (10.7)	P = 0.032
Angioectasia, n (%)	2 (1.9)	3 (2.1)	P = 0.694
Bleeding spot, n (%)	2 (1.9)	2 (1.5)	P = 1.000
Hyperemia/erythema, n (%)	12 (11.3)	16 (12.2)	P = 0.832
Polyp, n (%)	4 (3.8)	6 (4.6)	P = 1.000
Tumor, n (%)	1 (0.9)	4 (3.1)	P = 0.384s

10 patients were diagnosed as gastric tumors by MCE. 4 patients were in the older group and 6 patients in the oldest group. 3 patients’ families disagreed with EGD because of their poor general condition. 7 patients were followed up by EGD. 3 cases were early gastric cancer (78 M,79 M,85 M) and the remaining 4 patients were pathologically diagnosed as gastric adenocarcinoma. (Table S1)

Nausea during the gastric examination occurred in 1 patient in the oldest group. The completion rate to the cecum within 8 h was 65.9%; MCE failed to reach the cecum within 8 h in 67 patients in both groups. All patients checked capsule retention test by detector during 2 weeks. 292 patients in both groups spontaneously excreted the capsule in/at two weeks. Capsule retention in the cecum occurred in one case (0.34%). The patient in the oldest group(95 F) did not follow up for capsule excretion after two weeks.

## Discussion

In this retrospective study, MCE procedures in patients ≥ 60 years old were reviewed. This is the first study assessing transit time and gastric visualization and cleanliness scores in the oldest patients (≥ 80 years) compared with the older patients (60–80 years old). Our results demonstrate that MCE is feasible and safe for non-invasive gastrointestinal examinations of patients up to 100 years old.

The oldest patients had longer ETT and GET compared with older patients. Esophageal liquid and viscous bolus clearance [15] were impaired and gastric emptying of liquids was prolonged in healthy elderly subjects [16].

ETTs in our study were longer than the transit times shown by Hui X et al. [17] and Song J et al. [18]. The differences in transit times may be due to patient age differences; patients in our study were older than the Hui and Song studies. A high-resolution impedance manometry study demonstrated that peristaltic vigor and clearance were reduced in individuals over 80 years old [15]. Bolus flow through the esophagogastric junction was also reduced in asymptomatic older individuals. Both ineffective esophageal bolus transport and reduced swallow-induced esophagogastric junction relaxation contribute to impaired bolus flow in older individuals [19].

The MCE capsules were steered through the pylorus under magnetic control in almost 80% of patients and the PTT was significantly shortened by magnetic steering in both groups; therefore, no differences in PTT were detected between the two groups. The SITTs in patients over 80 years old were slightly, but not significantly, longer compared with the SITTs in patients under 80 years. Furthermore, the SITTs in our study were longer than the SITTs reported in a study by Tsibouris et al. [20]. These differences may be due to differences in the examination indications and the older age of patients in our study; patients in the Tsibouris et al. study were examined due to occult bleeding (including iron deficiency anemia) and ranged in age from 80 to 92 years [20]. In our study, no differences in PEG preparation and SITTs were detected between the two age groups. Reports on the effects of PEG on SITT studies are conflicting. Decreased SITTs after PEG preparation were observed in one RCT study [21]. However, a meta-analysis showed that PEG did not affect the SITT [22].

Gastric visualization and cleanliness in the oldest patients were inferior to visualization and cleanliness in the older patients. Therefore, patients over 80 years required longer times for gastric examination. Poor visualization in the two oldest patients was due to food debris. Elderly patients have more food residue, have worse transit capacity, [1] and take multiple medications, which may contribute to the poor visualization and cleanliness in these patients. Gastric diseases were risk factors for poor gastric cleanliness in our previous study [23]. In this study, the oldest patients had more examination indications (e.g., GI bleeding and dyspepsia) and inferior cleanliness scores compared with the older patient group. Furthermore, visualization in the fundus and body was slightly inferior in the oldest patients. Poor MCE visibility in the upper stomach was probably due to mucus and bubbles retained in the deep mucosal folds at the greater curvature [3, 8]. Poor visibility may also be caused by reduced proximal mucosa-detergent contact time due to the effects of gastric peristalsis and gravity [8].

Except for gastrointestinal injury assessment, gastrointestinal bleeding and dyspepsia were the most frequent indications for MCE in our study. Muhammad et al. [24] and Riccardo U et al. [25]demonstrated that the diagnostic yield of capsule endoscopy progressively increased with advancing age and was the highest in patients over 85 years. In our study, the diagnostic yield in patients over 80 was also higher, mainly due to a high incidence of intestinal ulcers and erosions. Intestinal mucosal erosions significantly increased with the intake of oral antithrombotic agents [26] among the oldest patients. Feng G et al. [26] showed that gastric ulcers and erosions were not significantly higher in the oldest group compared with the older group.

This study was limited by the failure rate of the capsule endoscopy to reach the cecum within 8 h in approximately 30% of patients in both groups, which is a higher incidence than most published studies [20]. According to our study protocol, check suits were removed and examinations were terminated at 8 h. Studies with prolonged examination times should be performed in the future. The other limitation is the lack of urea breath tests data and state of atrophic gastritis in previous MCE. As the MCE diagnostic value of HP and state of gastritis increases, we have collected urea breath tests data and specific mucosal findings recently. In the future, we will concentrate on HP infection status and classification of gastritis.

## Conclusion

This retrospective study compared transit times and gastric preparation between patients over 80 years and patients 60–80 years old. We confirmed the benefits and safety of MCE for aging patients. For the oldest patients with gastrointestinal diseases and poor motility, the gastric examination may take longer and strategies for better gastric visibility should be considered.

### Electronic supplementary material

Below is the link to the electronic supplementary material.

Supplementary Material 1

Supplementary Material 2

## Data Availability

The datasets generated during the current study are not publicly available due to the confidentiality of human subjects but are available from the corresponding author upon reasonable request.
