# Supplementary material for: Magnetic-controlled capsule endoscopy performance in aging patients
Source: BMC Gastroenterol. 2023 Aug 11;23:277. doi: 10.1186/s12876-023-02914-0 (PMC10422704; doi:10.1186/s12876-023-02914-0)
Supplement: Supplementary file 1 — Supplementary Material 1 [file 12876_2023_2914_MOESM1_ESM.docx]

Table S1. Details of gastric tumors

| No. | Age | Sex | Indications | Positve findings | Early gastric cancer | EGD | EGD outcomes | |
| --- | --- | --- | --- | --- | --- | --- | --- | --- |
| 1 | 70 | Male | Physical examination | Antrum tumor and bleeding | No | Yes | Gastric adenocarcinoma | |
| 2 | 76 | Male | GI injury assessment | Angulus tumor | No | No | NA | |
| 3 | 78 | Male | Physical examination | Antrum tumor | Yes | Yes | HGIN+  intramucosal carcinoma | |
| 4 | 79 | Male | Dyspepsia | Angulus Ulcer | Yes | Yes | HGIN+  intramucosal carcinoma |  |
| 5 | 85 | Male | Weight loss | Antrum tumor | Yes | Yes | HGIN+LGIN | |
| 6 | 88 | Male | GI injury assessment | Angulus tumor | No | Yes | Gastric adenocarcinoma | |
| 7 | 89 | Male | Dyspepsia | Antrum tumor | No | Yes | Gastric adenocarcinoma | |
| 8 | 90 | Female | GI bleeding | Angulus Ulcer and antrum tumor | No | No | NA | |
| 9 | 91 | Male | Poor appetite | Body tumor | No | Yes | Gastric adenocarcinoma | |
| 10 | 95 | Male | GI bleeding | Antrum tumor | No | No | NA | |

HGIN, high-grade gastric intraepithelial neoplasia, LGIN, low-grade gastric intraepithelial neoplasia, NA, not available.
