# Supplementary material for: Magnetic-controlled capsule endoscopy performance in aging patients
Source: BMC Gastroenterol. 2023 Aug 11;23:277. doi: 10.1186/s12876-023-02914-0 (PMC10422704; doi:10.1186/s12876-023-02914-0)
Supplement: Supplementary file 2 — Supplementary Material 2 [file 12876_2023_2914_MOESM2_ESM.docx]

Table S2. Comparison between the visualisation of preparation with PEG and SP

|  | PEG (N=53) | SP (N=240) | P value |
| --- | --- | --- | --- |
| Total gastric cleanliness scores |  |  |  |
| ALL patients (N=293) | 21.0(19.0,22.0) | 21.0(20.0,22.0) | 0.951 |
| The older group (N=165) | 21.0(19.0,21.3) | 21.0(20.0,22.0) | 0.704 |
| The oldest group (N=128) | 21.0(19.5,22.0) | 20.0(19.0,21.0) | 0.603 |
| Total gastric visualization scores |  |  |  |
| ALL patients (N=293) | 18.0(16.0,18.0) | 18.0(16.0,18.0) | 0.927 |
| The older group(N=165) | 18.0(16.0,18.0) | 18.0(17.0,18.0) | 0.918 |
| The oldest group(N=128) | 18.0(16.5,18.0) | 18.0(16.0,18.0) | 0.997 |

PEG, polyethylene glycol, SP, simethicone/pronase.
